# Anatomical and morphometrical study of middle ear ossicles in 2 to 3-month-old Makouei sheep fetuses

**Published:** 2017-09-15

**Authors:** Naeimeh Simaei, Farhad Soltanalinejad, Gholamreza Najafi, Ali Shalizar Jalali

**Affiliations:** Department of Basic Sciences, Faculty of Veterinary Medicine, Urmia University, Urmia, Iran.

**Keywords:** Anatomy, Fetus, Middle ear, Ossicle, Sheep

## Abstract

The middle ear ossicles are important due to transmission of sound to the inner ear leading to sound understanding. The aim of present study was to determine the anatomical and morphometrical aspects of middle ear ossicles in Makouei sheep fetuses. For this experimental study, eight sheep fetuses at the age of 2 to 3 months were provided from public slaughterhouse; their middle ear ossicles were removed from tympanic cavity subsequently and assessed anatomically using stereomicroscope. For statistical analysis, one-way ANOVA and Tukey’s post-hoc test were used. The results showed that rostral process of malleus doesn’t exist, but an osseous lamina extending to the tympanic bulla and tympanic ring is located in this place. Moreover, lenticular bone and muscular process of stapes weren’t found. These findings were similar to the other animal’s ossicles anatomical features, but there were also some differences that can be useful for study of these ossicles developmental evolution.

## Introduction

Ear is an organ that exhibits complicated organization in all organisms in terms of its anatomical and functional features.^1^ Overall, in addition to receiving sounds and auditory perception, ear plays an important role in body balance.^2^ The ear has three subdivisions: external ear, middle ear and internal ear.^3^ The middle ear is housed in the temporal bone and the small air-filled space known as tympanic cavity. The transmission of sound waves across the tympanic cavity is mediated by three auditory ossicles including malleus, incus and stapes in a lateromedial sequence.^4^ These ossicles are placed in the middle part of tympanic cavity^5^ and form a chain across the tympanic cavity from tympanic membrane to fenestra vestibuli.^6^ The malleus is the most lateral and the largest of the three bones, consists of head, neck and handle attaching to the tympanic membrane.^7^ The incus articulates with the head of the malleus by means of its body and the latter articulates with the head of the stapes through its long crus.^5^ The stapes is attached to the edge of the oval window.^5^ Several anatomical and morphological studies have been conducted to examine human and some animals middle ear ossicles. Previous reports in human have revealed the developmental characteristics of fetal ear stapes,^8^ morphology and evolution of the middle ear ossicles ossification^8^^-^^15^ and morphometrical and morphological variations of middle ear ossicles in newborns.^16^ Morphological observations have indicated that incus is the most stable and stapes is the most variable of all. Based on anatomical studies of auditory ossicles, lenticular bone is present in New Zealand rabbits^5^ and mice,^17^ whereas it is not present in sheep.^18^ It was found that similar to human beings, sheep middle ear has a typical respiratory epithelium.^18^ Middle ear ossicles have an important role in transmission and understanding of sounds. There is no anatomical and morphometrical report about these ossicles in Makouei sheep fetuses.

In line with that, the present study was undertaken to explore the anatomical and morphometrical features of auditory ossicles in Makouei sheep fetuses. 

## Materials and Methods

Eight sheep fetuses at the age of 2 to 3 months were collected from public slaughterhouse and examined in this study. Fetal age was determined using the following formula: 

*X = 2.10 (Y+17)*

where, *X* is the developmental age in days and *Y* is the fetus crown to rump length (CRL) in centimeter.^19^ Fetuses were fixed in 10% formalin solution following age determination. Then, the middle ear ossicles were removed from tympanic cavity and assessed anatomically using stereomicroscope (Model SZX-ILLB200; Olympus Co., Tokyo, Japan). Further, anatomical parameters including length and width of the malleus handle, height and base size of the malleus muscular process, lateral process height, length of ossicular lamina, length and width of long and short limbs of the incus, length, width and thickness of the incus body, length and width of the rostral and caudal crura of the stapes and large and small diameters of the intercrural foramen of the stapes were measured. For statistical analyses, one-way ANOVA using SPSS software (Version 23.0, IBM Company, Chicago, USA) and Tukey’s post-hoc test were used and the value of *p* < 0.05 was considered as the criterion for statistical significance.

## Results

**Fig. 1:**
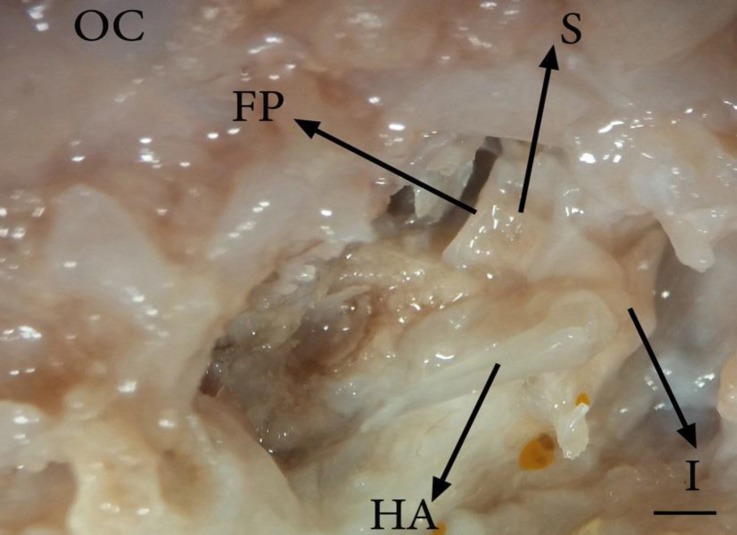
Ossicular chain connection in 86-day-old sheep fetus (ventral view); HA: Handle of malleus; I: Incus; S: Stapes; FP: Foot plate and OC: Occipital condyle (Bar = 1 mm

**Fig. 2:**
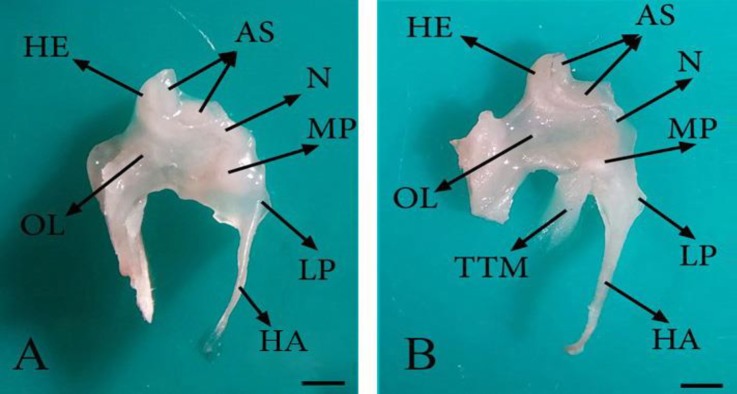
Malleus in 82- (A) and 84-day-old (B) sheep fetuses; HE: Head of malleus; AS: Articular surface; N: Neck; LP: Lateral process; HA: Handle of malleus; MP: Muscular process; OL: Ossicular lamina and TTM: Tensor tympani muscle (Bar = 1 mm

**Fig. 3:**
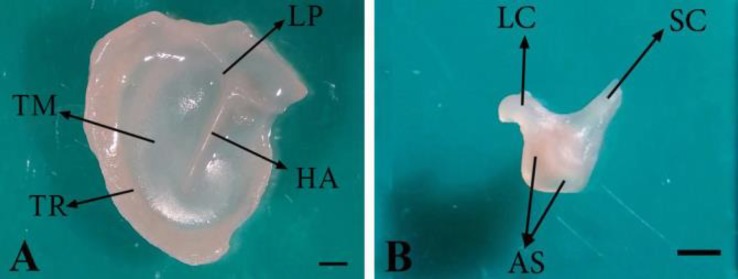
**A)** Position of the handle of malleus embedded within the tympanic membrane in 74-day-old sheep fetus; HA: Handle of malleus; LP: Lateral process of malleus; TM: Tympanic membrane and TR: Tympanic ring. **B)** Incus in 76-day-old sheep fetus; LC: Long crus; SC: Short crus and AS: Articular surface (Bar = 1 mm

Anatomical examinations in all dissected fetuses showed that tympanic cavity contains three auditory ossicles including malleus, incus and stapes. The malleus is the most lateral and the largest ossicle and the stapes is the innermost and the smallest of all (Fig. 1). The malleus consists of a handle, a head, a neck and three processes (Fig. 2). In the current study, the base of malleus handle was three-sided in cross-section. The lateral side of handle, connected to the medial surface of tympanic membrane, was thinner than the others. In addition, the handle of malleus decreased in diameter towards its distal extremity without any curvature (Figs. 1, 2 and 3A). The handle was connected to the head with a short neck. The head of malleus was nearly oval and articulated with the body of incus via its articular surface. It had also a caudo-medial direction (Fig. 2). The lateral process of the malleus was the most dorsal attachment of the handle to the tympanic membrane. This process as a triangular projection was extended from the root of the handle causing a bulge in the tympanic membrane (Figs. 2 and 3A). 

The conical muscular process was located near the proximal end of malleus handle’s medial border and attached to the tendon of the tensor tympani muscle (Fig. 2). Rostral process of malleus didn’t exist, but an osseous lamina extending to the tympanic ring was located in this place (between the head and muscular process of malleus), (Fig. 2). The incus as a second ossicle was located between two others (Fig. 1) and divided into a body and short and long limbs (Fig. 3B). The body was convex and cube-shaped and articulated with the head of malleus through articular surface. The limbs were originated from body divergently. The short limb was developed caudally and the long limb was turned ventro-medially in epitympanic recess and articulated with the head of stapes in the absence of lenticular bone (Figs. 1 and 3B).

**Fig. 4:**
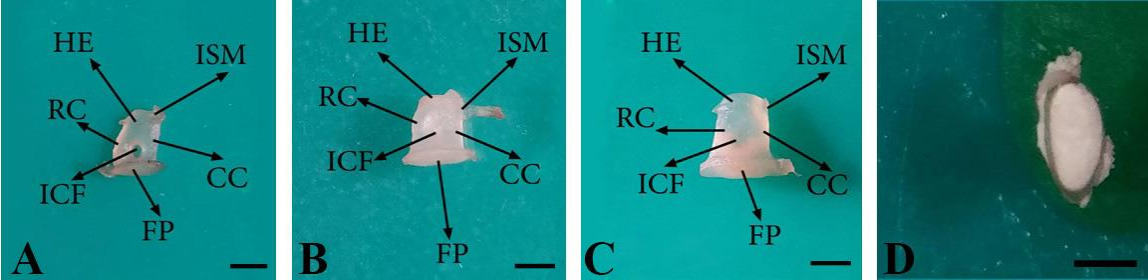
Stapes in 74-(A), 76-(B) and 80-day-old (C) sheep fetuses and foot plate of stapes in 89-day-old sheep fetus (D); HE: Head of malleus; ISM: Insertion of stapedius muscle; RC: Rostral crus; CC: Caudal crus; ICF: Intercrusal foramen and FP: Foot plate (Bar = 1 mm

The assessments showed that stapes is the innermost and the smallest ossicle (Fig. 1) comprising a head, a base, a neck, a footplate, two crura (one with rostral and the other with caudal placements) and intercrural foramen. However, the muscular process of stapes was absent. There was a distinctive surface at the junction of the head and caudal crus of stapes as a stapedius muscle insertion (Figs. 4A, B, C). The head of stapes had an articular surface making a joint with long limb of incus. Stapes was observed in different shapes from rectangle (Fig. 4B) to trapezoid (Fig. 4C). The stapes crura were seen in a symmetrical (Fig. 4C) and/or asymmetrical manner (Figs. 4A and 4B). Moreover, stapes intercrural foramen was observed as an oval-shaped hollow (Fig. 4A) and/ or very tiny recess (Figs. 4B and 4C). The crura were connected by a flattened and oval-shaped base at their distal extremities, called foot plate (Fig. 4D). The foot plate was articulated with vestibular window (Fig. 1).

The anatomical parameters of malleus are shown in Table 1. There were significant differences between 84-day-old and 74- and 76-day-old fetuses regard to the length of malleus handle (*p* < 0.05), whereas there were no significant differences among 84- and 81-day-old fetuses (*p* > 0.05). Moreover, there were significant differences between 81-day-old and 74- and 76-day-old fetuses regard to the length of malleus handle (*p* < 0.05).

The anatomical features of incus are presented in Table 2. Length of the long limb of incus in 84-day-old fetuses was significantly higher than 74-, 76- and 81-day-old fetuses (*p* < 0.05). In addition, there were significant differences between 84-day-old and 74- and 76-day-old fetuses regard to the thickness of incus body (*p* < 0.05), but there were no significant differences between 84- and 81-day-old fetuses (*p* > 0.05). Additionally, there were significant differences between 81- and 74- and 76-day-old fetuses (*p* < 0.05).

Anatomical characteristics of stapes are presented in Table 3. The data revealed that these parameters relatively increase with age increasing (*p* > 0.05) and the rostral crus of stapes is longer than caudal one. 

**Table 1:** Morphometrical parameters (Mean ± SE) of malleus in 2- to 3-month-old Makouei sheep fetuses

**Age of fetus (days)**	**Handle length** ** (mm)**	**Handle width** ** (mm)**	**Muscular process height** ** (mm)**	**Muscular process base size** ** (mm)**	**Lateral process height** ** (mm)**	**Ossicular lamina length** ** (mm)**
**74**	3.37 ± 0.05^a^	0.29 ± 0.06	0.59 ± 0.05	0.44 ± 0.03	0.44 ± 0.04	1.49 ± 0.03
**76**	3.88 ± 0.10^a^	0.29 ± 0.01	0.59 ± 0.11	0.44 ± 0.13	0.37 ± 0.02	1.49 ± 0.06
**81**	4.47 ± 0.05^b^	0.37 ± 0.03	0.67 ± 0.06	0.44 ± 0.01	0.58 ± 0.07	1.38 ± 0.09
**84**	4.77 ± 0.12^bc^	0.37 ± 0.07	0.74 ± 0.02	0.59 ± 0.02	0.59 ± 0.02	1.73 ± 0.11

abc Different superscripts indicate significant differences in each column (*p *< 0.05).

**Table 2.** Morphometrical parameters (Mean ± SE) of incus in 2- to 3-month-old Makouei sheep fetuses

**Age of fetus (days)**	**Long limb length** ** (mm)**	**Long limb width** ** (mm)**	**Short limb length** ** (mm)**	**Short limb width** ** (mm)**	**Body length** ** (mm)**	**Body width** ** (mm)**	**Body thickness** ** (mm)**
**74**	1.34 ± 0.04^a^	0.44 ± 0.01	0.89 ± 0.03	0.29 ± 0.04	1.19 ± 0.01	1.34 ± 0.02	0.65 ± 0.04^a^
**76**	1.49 ± 0.06^a^	0.44 ± 0.04	0.89 ± 0.01	0.29 ± 0.01	1.21 ± 0.13	1.34 ± 0.01	0.89 ± 0.19^a^
**81**	1.64 ± 0.07^a^	0.46 ± 0.07	1.34 ± 0.01	0.52 ± 0.06	1.24 ± 0.09	1.64 ± 0.03	1.34 ± 0.03^bc^
**84**	1.94 ± 0.03^b^	0.59 ± 0.04	1.49 ± 0.05	0.57 ± 0.15	1.34 ± 0.07	1.49 ± 0.09	1.49 ± 0.03^bc^

abc Different superscripts indicate significant differences in each column (*p *< 0.05).

**Table 3.** Morphometrical parameters (Mean ± SE) of stapes in 2- to 3-month-old Makouei sheep fetuses

**Age of fetus (days)**	**Rostral limb length** ** (mm)**	**Rostral limb width** ** (mm)**	**Caudal limb length** ** (mm)**	**Caudal limb width** ** (mm)**	**Intercrural foramen large diameter** ** (mm)**	**Intercrural foramen small diameter** ** (mm)**
**74**	1.19 ± 0.05	0.29 ± 0.03	1.04 ± 0.10	0.29 ± 0.03	0.29 ± 0.01	0.29 ± 0.06
**76**	1.42 ± 0.06	0.43 ± 0.06	1.22 ± 0.13	0.33 ± 0.03	0.29 ± 0.07	0.27 ± 0.01
**81**	1.64 ± 0.05	0.52 ± 0.07	1.34 ± 0.06	0.34 ± 0.08	0.29 ± 0.04	0.29 ± 0.02
**84**	1.64 ± 0.12	0.50 ± 0.07	1.64 ± 0.03	0.37 ± 0.06	0.44 ± 0.05	0.29 ± 0.04

There were no significant differences between these data (*p *< 0.05).

## Discussion

There are few anatomical and morphometrical reports about middle ear ossicles in human and animals. It has been reported that the shape of malleus head is oval in hamsters,^20^ hemispherical or spherical in moles,^20^ nearly ovoid in bovine fetuses and flattened in cattle.^1^ In this study, the shape of malleus head was oval-shaped, the same as hamsters and the malleus head in sheep fetuses did not show evident flattening as reported for cattle. 

It was found that the handle of malleus curves slightly forward, its base cross-section is completely three-sided and the lateral side is wider than the others in New Zealand rabbits.^5^ The curvature of the malleus handle was not reported in human fetuses,^21^ while it curved slightly forward in newborns.^16^

In the present study, the handle of malleus had no curvature, which is in agreement with previous findings in human fetuses.^21^ In addition, similar to New Zealand rabbits, in sheep fetuses the cross-section of malleus handle was three-sided,^5^ but the lateral side was thinner than the others. It has been shown that the neck of malleus is not found in human fetuses^21^ and newborns^16^^,^^21^ and it is relatively long in New Zealand rabbits.^5^ In agreement with previous findings in mice and hamsters,^17^ in the present study, the handle was connected to the head with a neck, but it was short and not well-developed.

Reportedly, the lateral process of malleus is cone-shaped in hamsters ^20^ and it has a variety of shapes in human newborns,^16^ but it was observed as a triangular projection in the current study. It has been revealed that the rostral process of malleus is rose-thorn-shaped in adult ruminants,^22^ it is mostly embedded within the tympanic membrane in New Zealand rabbits^5^ and it is unclear and not well–developed in hamsters.^20^ Further, it has been indicated that the length of rostral process is variable and/or this process is quite long in human newborns.^16^ It has also been reported that the rostral process is the longest process of malleus in fetal life of human becoming shorter after birth.^23^ In the present research, the rostral process of malleus wasn’t found, but an osseous lamina extending to the tympanic ring was located in this place. 

It has been shown that the body of incus is oval-shaped in adult ruminants,^22^ but it was convex and cube-shaped in this study. It has been observed that two limbs of incus are emerged from the body at a right angle in bovine fetuses,^1^ but in the current study, the limbs were appeared from two different angles of body. On the other hand, limbs of incus were reported to have same length in adult sheep,^24^ but our findings showed that the long limb is longer than the other one. Previous studies have described that lenticular bone is present in hamsters,^20^ adult ruminants^22^ and New Zealand rabbits,^5^ but it was absent in sheep fetuses in the current study.

It has been reported that the stapes is stirrup-shaped in bovine fetuses^1^ and some rodents,^25^ rectangular in adult ruminants,^22^ triangular in hamsters^20^ and isosceles triangle-shaped in horses,^22^ but stapes was observed in different shapes from rectangle to trapezoid in this study. 

It has been shown that the crura of stapes have equal lengths in bovine fetuses and pigs;^8^ however, the caudal crus is longer than the rostral one in hamsters,^20^ humans and mole species.^2^^,^^26^ It was found that the stapes crura have symmetry and/or asymmetry in human newborns,^16^ which is in agreement with our findings. In accordance with our findings, the rostral crus of the stapes was reported to be longer than the caudal one in New Zealand rabbits.^5^

Previous studies have demonstrated that intercrural foramen of the stapes has various shapes in human fetuses and newborns^16^^,^^21^ and it is triangular in hamsters,^20^ but it was observed as an oval-shaped hollow and/or very tiny recess in the present study. It has been indicated that muscular process of stapes is present in adult ruminants^22^ and New Zealand rabbits,^5^ but our examinations revealed that it is absent in sheep fetuses like bovine ones. ^1^

It could be concluded that anatomical findings of auditory ossicles examinations in 2- to 3-month-old Makouei sheep fetuses are similar to the other animals ossicles anatomical features, but there are also some differences such as presence of an osseous lamina instead of malleus rostral process and absence of lenticular bone and muscular process of stapes. These findings can be useful for future studies of these ossicles developmental evolution. 
